# Supplementary material for: Group threat, political extremity, and collective dynamics in online discussions
Source: Sci Rep. 2023 Feb 7;13:2206. doi: 10.1038/s41598-023-28569-1 (PMC9905083; doi:10.1038/s41598-023-28569-1)
Supplement: Supplementary file 1 — Supplementary Information. [file 41598_2023_28569_MOESM1_ESM.pdf]

N. Gizem Bacaksizlar Turbic and Mirta Galesic: Group Threat, Political Extremity, and Collective Dynamics in Online Discussions

Supplementary Material

Table S1. Commenter, comment, and article counts for articles published by Mother Jones, The Atlantic, The Hill, and Breitbart, during the month before and the month after different events.

			Month Before			Month After		
**Event**	**Date**	**News Site**	**Commenters**	**Comments**	**Articles**	**Commenters**	**Comments**	**Articles**
1. Orlando Attack	12-Jun-2016	Mother Jones	7,336	95,862	912	5,456	86,830	887
		The Atlantic	13,338	185,918	2,153	12,359	181,401	2,217
		The Hill	35,203	1,480,553	4,759	31,596	1,523,329	4,743
		Breitbart	46,966	2,017,814	8,283	50,800	2,052,007	8,077
2. Brexit Referendum	23-Jun-2016	Mother Jones	5,948	93,130	468	5,103	77,151	453
		The Atlantic	13,223	188,074	1,320	11,829	171,601	1,277
		The Hill	33,595	1,538,196	3,697	34,414	1,574,227	3,436
		Breitbart	48,695	2,017,959	20,956	52,226	2,231,633	27,917
3. United States Election	8-Nov-2016	Mother Jones	4,517	251,042	2,137	5,021	249,643	1,956
		The Atlantic	10,738	619,849	5,152	9,723	517,866	4,254
		The Hill	35,474	1,818,410	5,732	37,201	4,459,860	13,713
		Breitbart	59,449	7,946,536	22,058	66,565	8,774,603	24,405
4. Presidential Inauguration	20-Jan-2017	Mother Jones	4,255	75,229	730	5,420	86,183	863
		The Atlantic	8,974	156,660	1,751	12,050	194,933	1,908
		The Hill	30,681	1,536,162	4,877	41,080	1,974,873	5,883
		Breitbart	61,052	2,701,699	9,199	72,863	3,495,675	10,159
5. Charlottesville Rally	11-Aug-2017	Mother Jones	3,853	84,214	875	3,316	77,417	780
		The Atlantic	10,147	215,257	2,071	10,915	228,683	1,839
		The Hill	36,147	2,091,593	6,110	32,557	1,992,493	5,460
		Breitbart	54,180	3,415,882	8,824	60,607	3,791,626	9,009
6. Las Vegas Shooting	1-Oct-2017	Mother Jones	3,085	65,009	771	3,225	64,423	801
		The Atlantic	7,860	174,900	1,873	9,147	218,059	1,870
		The Hill	33,258	1,826,011	5,949	38,152	2,278,911	6,507
		Breitbart	57,811	3,541,065	9,072	60,258	3,817,425	9,458
7. United States Election	6-Nov-2018	Mother Jones	2,343	199,326	1,935	2,697	175,379	1,840
		The Atlantic	-	-	-	-	-	-
		The Hill	30,285	7,902,881	14,733	27,753	8,058,085	15,295
		Breitbart	58,928	10,613,517	23,867	55,647	8,842,971	19,236

Table S2. The top five events from the U.S. news according to Google Trends from 2016 - 2018 [34].

Rank	2016 U.S. News	2017 U.S. News	2018 U.S. News
1	Olympics	Hurricane Irma	World Cup
2	Election	Las Vegas Shooting	Hurricane Florence
3	Orlando Shooting	Solar Eclipse	Mega Millions
4	Brexit	Hurricane Harvey	Election Results
5	Zika Virus	Bitcoin Price	Hurricane Michael

Table S3. Correlation coefficients and bootstrapped confidence intervals for trends shown in Figures 2, A5-A9, jointly for the two left-leaning sites (Mother Jones and The Atlantic) and the moderate and right-leaning sites (The Hill and Breitbart).

	**Correlation of survey ratings of threat and network indices of:**			
	**Skewness of in-degree**	**Skewness of Page Ranks**	**p(independent commenters)**	**p(connected components)**
		**Left-oriented news sites (Mother Jones and The Atlantic)**		
Ingroup hurt more than, or same as, outgroup after the event	0.72 [0.31, 0.87]	0.76 [0.39, 0.89]	0.3 [-0.41, 0.77]	0 [-0.66, 0.61]
Ingroup felt more threatened than outgroup after the event	0.55 [0.09, 0.82]	0.62 [0.08, 0.85]	0.23 [-0.57, 0.75]	-0.06 [-0.72, 0.56]
Ingroup felt more threatened after than before the event	0.54 [0.11, 0.78]	0.61 [0.14, 0.83]	0.33 [-0.48, 0.83]	-0.07 [-0.75, 0.55]
Personal feeling of threat after the event	0.2 [-0.48, 0.62]	0.29 [-0.41, 0.67]	0.33 [-0.26, 0.71]	-0.23 [-0.74, 0.3]
Personally felt more threatened after than before the event	0.36 [-0.26, 0.69]	0.43 [-0.26, 0.75]	0.23 [-0.47, 0.72]	-0.19 [-0.75, 0.39]
Overall, people felt more threatened after than before the event	0.24 [-0.33, 0.61]	0.31 [-0.25, 0.63]	0.2 [-0.38, 0.6]	-0.39 [-0.82, 0.11]
		**Moderate- and right-oriented news sites (The Hill and Breitbart)**		
Ingroup hurt more than, or same as, outgroup after the event	0.78 [0.5, 0.95]	0.5 [0.08, 0.79]	-0.76 [-0.92, -0.47]	-0.6 [-0.81, -0.05]
Ingroup felt more threatened than outgroup after the event	0.7 [0.2, 0.95]	0.41 [-0.18, 0.75]	-0.65 [-0.9, -0.28]	-0.45 [-0.75, 0.06]
Ingroup felt more threatened after than before the event	0.57 [-0.04, 0.88]	0.29 [-0.37, 0.69]	-0.59 [-0.84, -0.17]	-0.42 [-0.76, 0.07]
Personal feeling of threat after the event	0.14 [-0.41, 0.51]	-0.03 [-0.63, 0.42]	-0.3 [-0.68, 0.16]	-0.1 [-0.53, 0.36]
Personally felt more threatened after than before the event	0.27 [-0.39, 0.62]	0.06 [-0.56, 0.55]	-0.43 [-0.79, 0.2]	-0.32 [-0.78, 0.36]
Overall, people felt more threatened after than before the event	0.39 [-0.24, 0.73]	0.17 [-0.54, 0.63]	-0.45 [-0.8, 0.1]	-0.13 [-0.61, 0.39]

Table S4. Percentage of commenters on each site who commented on other sites at least once.

Of all commenters who comment mostly on ..	. . . percentage who also commented on other site at least once:			
	Mother Jones	The Atlantic	The Hill	Breitbart
Mother Jones	100%	13%	13%	9%
The Atlantic	5%	100%	8%	6%
The Hill	8%	10%	100%	28%
Breitbart	4%	5%	18%	100%

Table S5. Percentage of comments on other sites, posted by commenters on each site.

Of all commenters who comment mostly on ..	. . . percentage who also commented on other site at least once:			
	Mother Jones	The Atlantic	The Hill	Breitbart
Mother Jones	91%	4%	3%	2%
The Atlantic	1%	95%	2%	2%
The Hill	1%	1%	90%	8%
Breitbart	0%	1%	3%	96%

Note: The main site of a commenter was determined as the site at which they posted most comments.

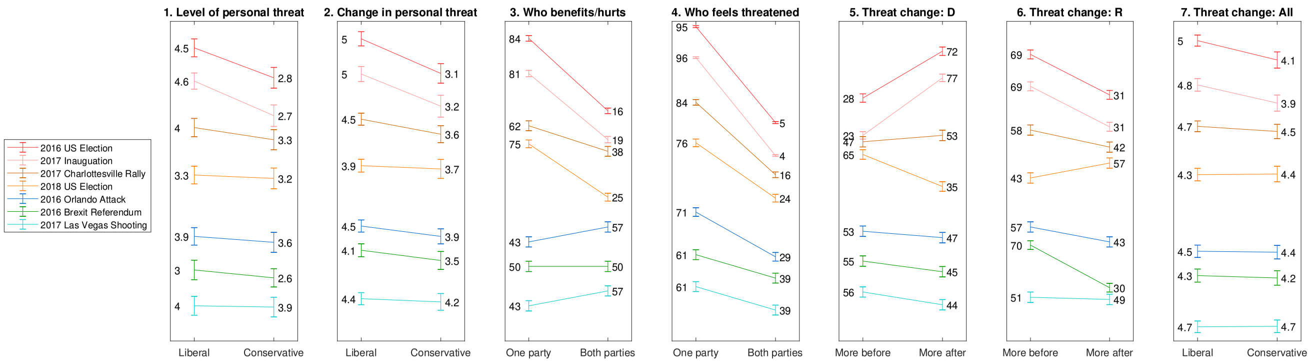

Figure S1. Perceived threat of societal events to different groups, for events hypothesized to be specific group threats (warm colors) and unspecific group threats (cold colors). See details in Method: Societal events. Questions in each panel were: 1. How threatened did you personally feel in the month after this event? *(1-Not at all threatened...7-Extremely threatened)*, 2. In the month after the event, did you feel more or less threatened compared to the month before the event? *(1-I felt much less threatened...7-I felt much more threatened)*, 3. In your view, did the event clearly benefit Democrats at the expense of Republicans, Republicans at the expense of Democrats, or were both groups affected similarly? *(The event benefited Democrats at the expense of Republicans; The event benefited Republicans at the expense of Democrats; The event benefited both Democrats and Republicans; The event hurt both Democrats and Republicans)*, 4. Who do you think felt more threatened in the month after this event, Democrats or Republicans? *(1-Democrats felt much more threatened...7-Republicans felt much more threatened)*, 5. Thinking now of Democrats, do you think they felt more or less threatened in the month after the event compared to the month before this event? *(1-Democrats felt much less threatened...7-Democrats felt much more threatened)*, 6. Thinking now of Republicans, do you think they felt more or less threatened in the month after the event compared to the month before this event? *(1-Republicans felt much less threatened...7-Republicans felt much more threatened)*, 7. Thinking now of all people in the U.S., independently of their political extremity: In the month after the event, did most people feel more or less threatened compared to the month before the event? *(1-Most people felt much less threatened...7-Most people felt much more threatened)*. Confidence intervals comprise on average +/- 4% of the mean of each rating, suggesting a strong agreement among participants about how threatened different groups should be - perhaps not surprising in today's extremely polarized political climate.

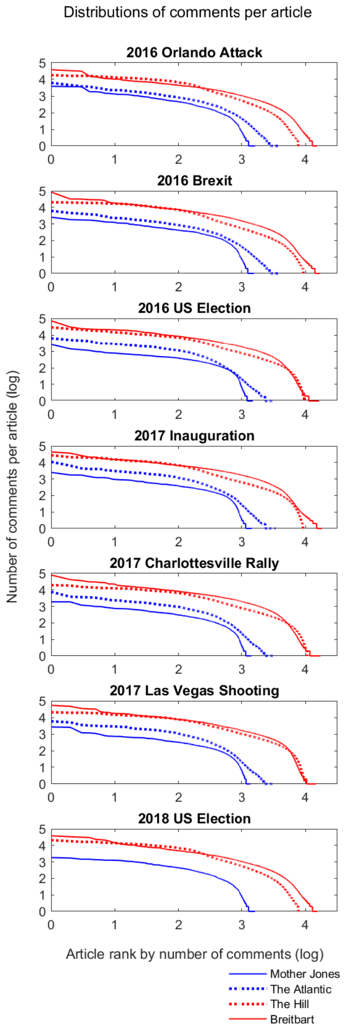

Figure S2. Distributions of the number of comments per article for different websites and events.

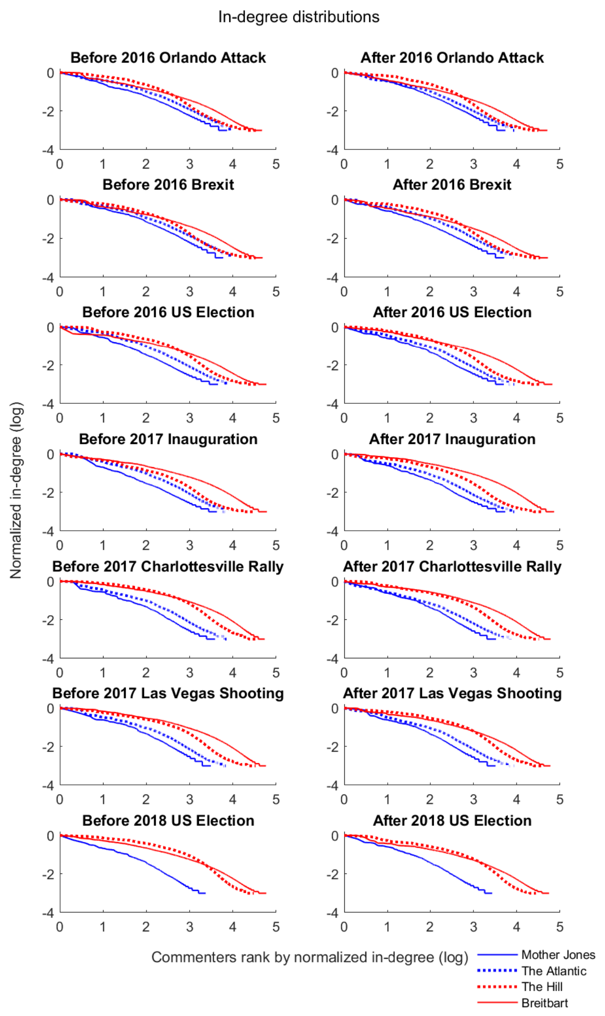

Figure S3. Distributions of commenters' in-degrees before and after important events.

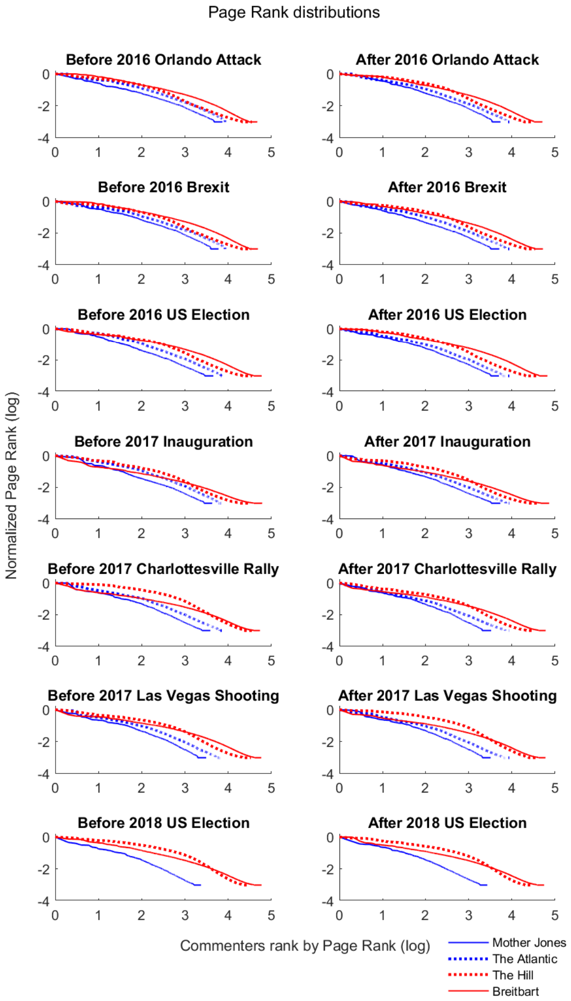

Figure S4. Distributions of commenters' Page Ranks before and after important events.

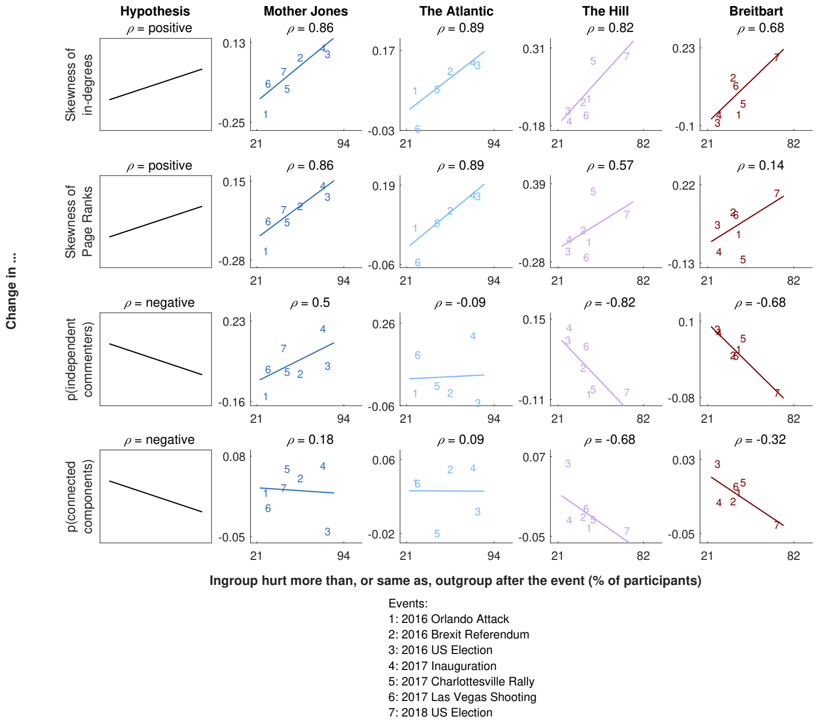

Figure S5. Changes in network measures of inequality from before to after events rated as more or less threatening to the ingroups on different news sites. The ratings (x-axis) are summarized as the percentage of participants who estimated that the event hurt the ingroup (Democrats for Mother Jones and The Atlantic, Republicans for The Hill and Breitbart) the same or more than the outgroup (survey question 3, see Figure S1). Rows show results for different measures of inequality of attention (y-axis), on the individual (the first two rows) and the network level (the second two rows). The first column shows the hypothesized median patterns of results, and the other columns show results for different sites. Colored numbers in each plot correspond to different events (see legend), and patterns are summarized by least-squares fitted lines across events and the Spearman ρ correlation coefficient above each plot.

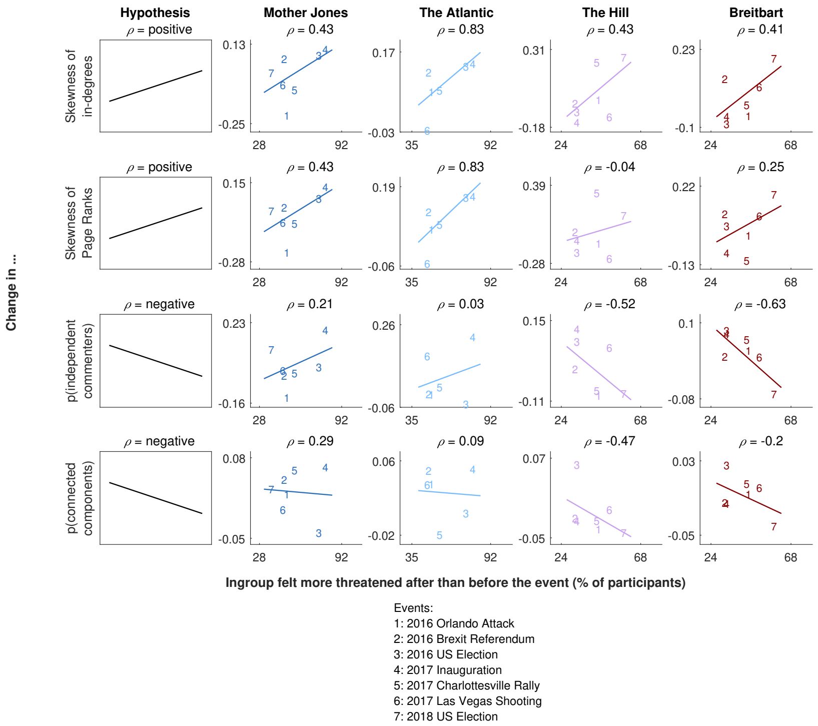

Figure S6. Changes in network measures of inequality from before to after events rated as more or less threatening to the ingroups on different news sites. The ratings (x-axis) are summarized as the percentage of participants who estimated that the ingroup (Democrats for Mother Jones and Atlantic, Republicans for The Hill and Breitbart) felt more threatened in the month after the event compared to the month before the event (5 or more on the 7-point scale, see Figure S1, survey questions 5 and 6). Rows show results for different measures of inequality of attention (y-axis), on the individual (the first two rows) and the network level (the second two rows). The first column shows the hypothesized median patterns of results, and the other columns show results for different sites. Colored numbers in each plot correspond to different events (see legend), and patterns are summarized by least-squares fitted lines across events and the Spearman ρ correlation coefficient above each plot.

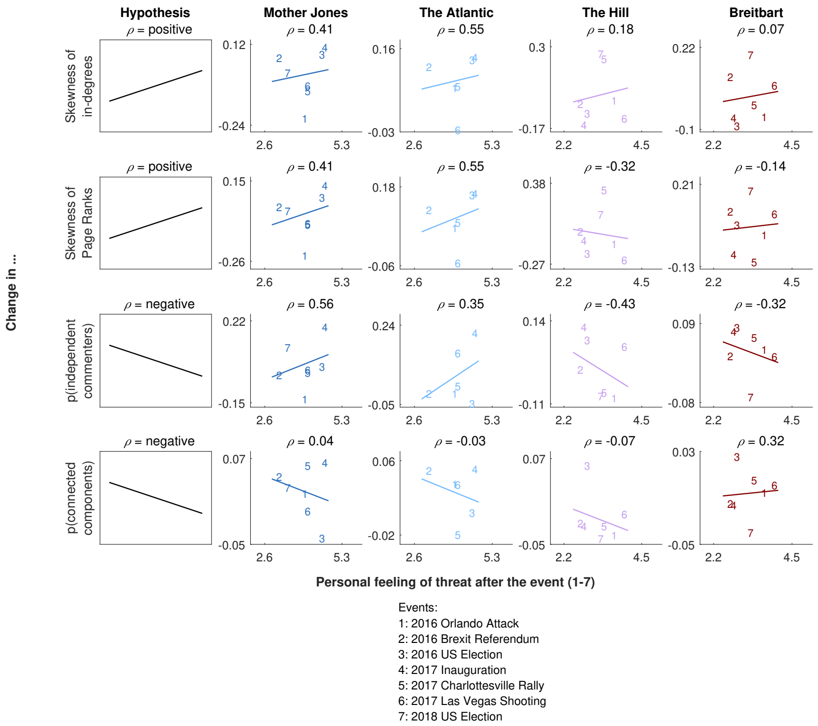

Figure S7. Changes in network measures of inequality from before to after events rated as personally more or less threatening to our liberal (for Mother Jones and The Atlantic) or conservative participants (for The Hill and Breitbart). The ratings (x-axis) are on a scale from 1 to 7, where 7 is the most threat (see Figure S1, survey question 1). Rows show results for different measures of inequality of attention (y-axis) on the individual (the first two rows) and the network level (the second two rows). The first column shows the hypothesized median patterns of results, and the other columns show results for different sites. Colored numbers in each plot correspond to different events (see legend), and patterns are summarized by least-squares lines across events and the Spearman ρ correlation coefficient above each plot.

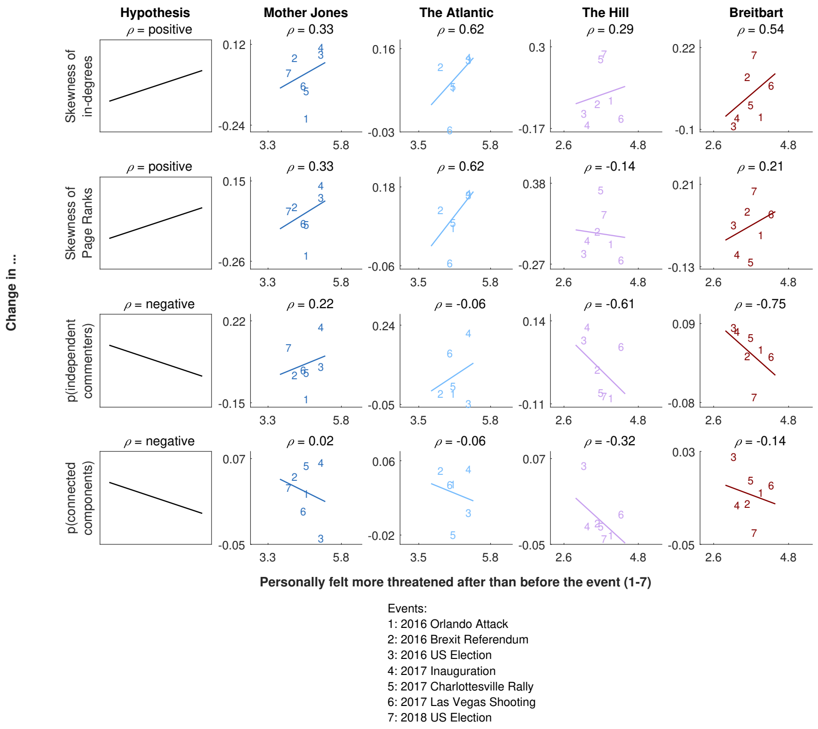

Figure S8. Changes in network measures of inequality from before to after events for which our liberal (for Mother Jones and Atlantic) or conservative participants (for The Hill and Breitbart) experienced more or less threat after compared to before the event. The ratings (x-axis) are on a scale from 1 to 7, where 7 is more threat after the event (see Figure S1, survey question 2). Rows show results for different measures of inequality of attention (y-axis) on the individual (the first two rows) and the network level (the second two rows). The first column shows the hypothesized median patterns of results, and the other columns show results for different sites. Colored numbers in each plot correspond to different events (see legend), and patterns are summarized by least-squares fitted lines across events and the Spearman ρ value above each plot.

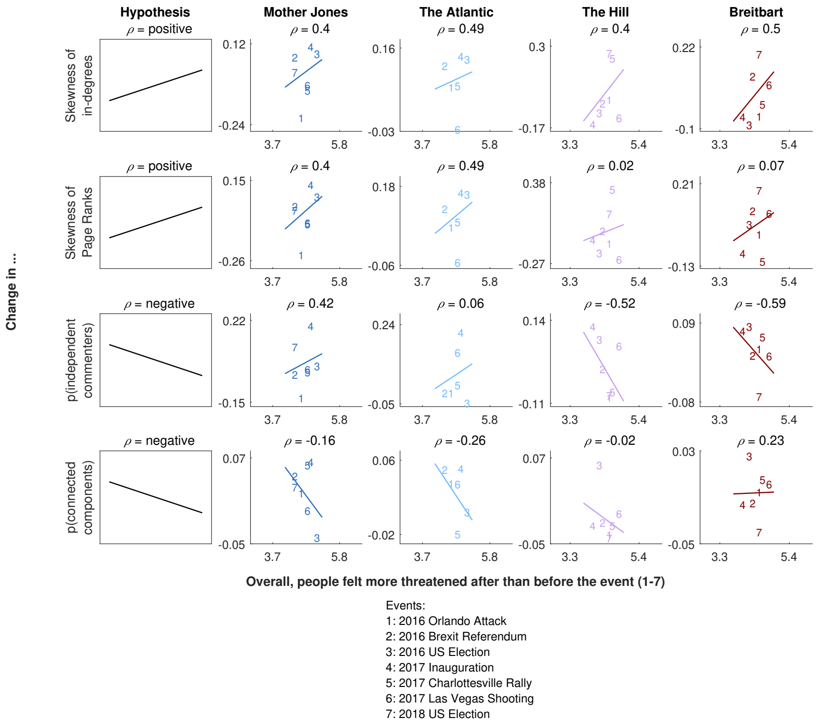

Figure S9. Changes in network measures of inequality from before to after events rated as more or less threatening to most people. The ratings (x-axis) are on a scale from 1 to 7, where 7 is more threat after the event for most people (see Figure S1, survey question 7). Rows show results for different measures of inequality of attention (y-axis), on the individual (the first two rows) and the network level (the second two rows). The first column shows the hypothesized median patterns of results, and the other columns show results for different sites. Colored numbers in each plot correspond to different events (see legend), and patterns are summarized by least-squares fitted lines across events and the Spearman ρ correlation coefficient above each plot.

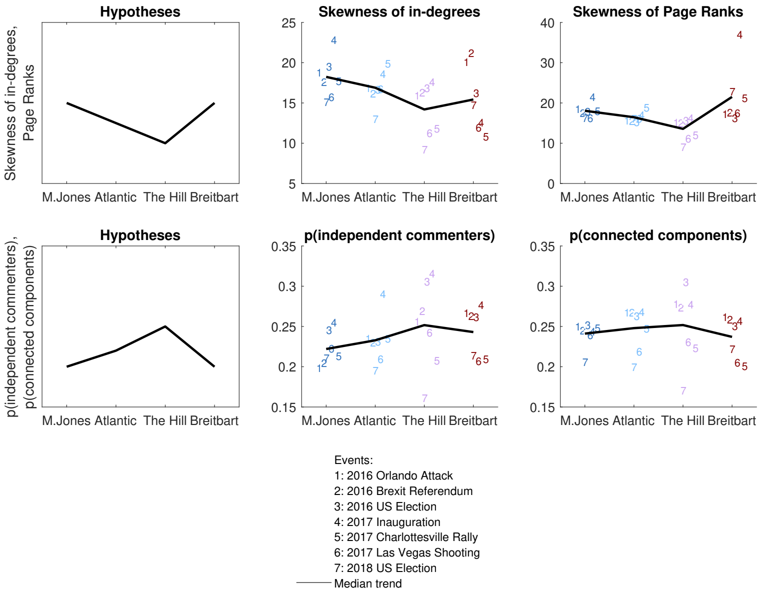

Figure S10. Differences in overall inequality of attention on different news websites, measured as the skew of centrality indices averaged across all commenters (compare with results for the top 1% commenters in Figure 2).

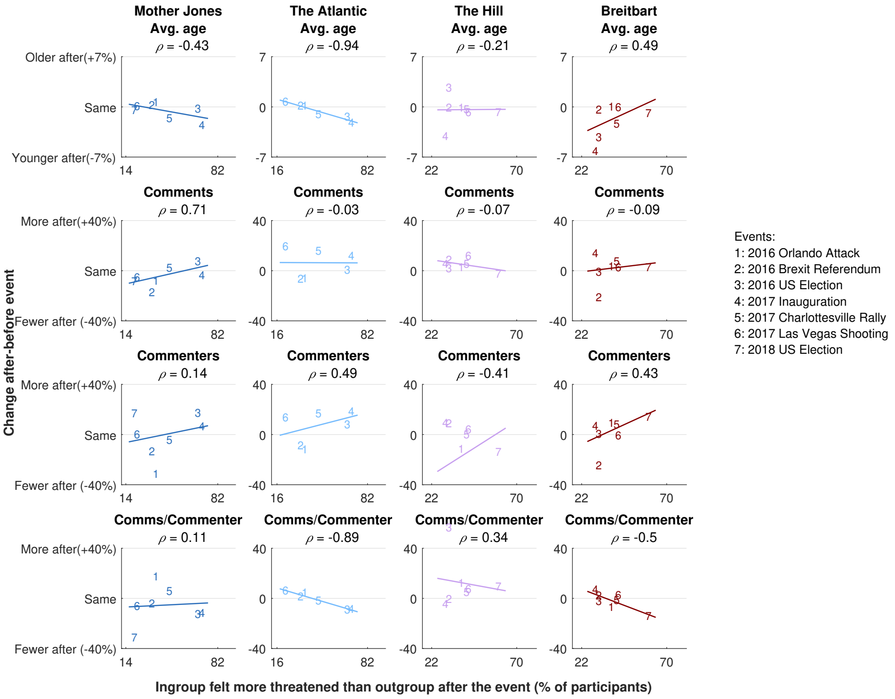

Figure S11. Descriptive measures of comments and commentators for events rated as more or less threatening to the ingroups on different news sites. The threat ratings (x-axis) are summarized as the percentage of participants who estimated that the ingroup on a given site (Democrats for Mother Jones and The Atlantic, Republicans for The Hill and Breitbart) felt more threatened by the event than the outgroup (survey question 4, Figure S1). The first row shows the percent change in the average "age" of commenters from before to after each event, where age is calculated as the difference in days between the event date and the date a commenter joined Disqus. The second row shows the percent change in the average number of comments per article from before to after each event. The third row shows the percent change in the average number of commenters per article from before to after each event. The fourth row shows the percent change in the ratio of comments to commenters from before to after each event. Colored numbers in each plot correspond to different events (see legend), and patterns are summarized by least-squares fitted lines across events and the Spearman ρ value above each plot.

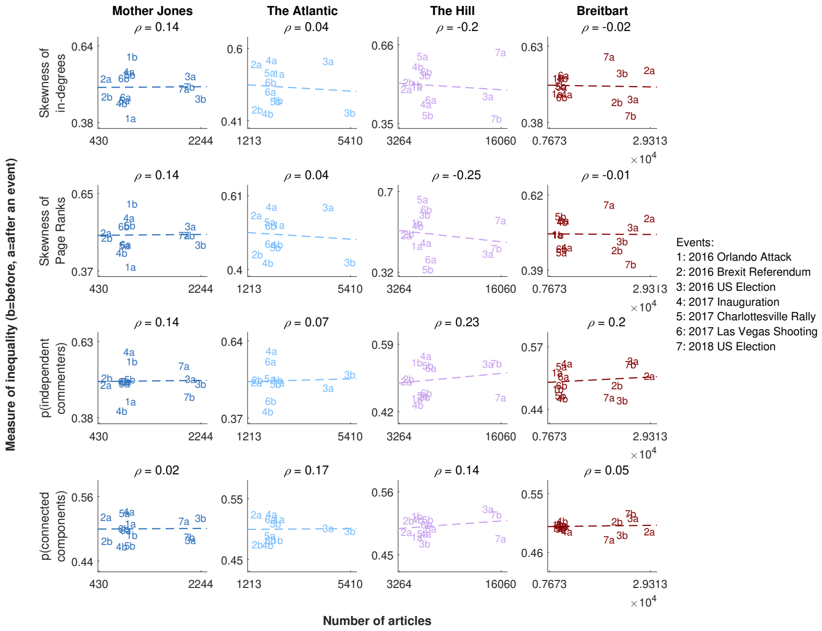

Figure S12. The relationship of different measures of inequality (y-axes) and the number of articles published before and after each event (x-axes). Colored numbers in each plot correspond to different events (see legend), and patterns are summarized by least-squares fitted lines across events and the Spearman ρ value above each plot.

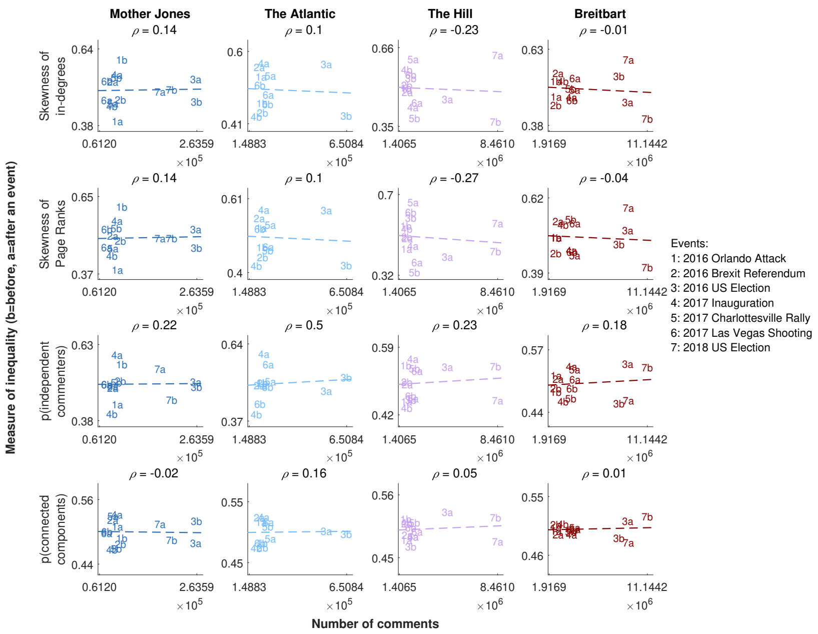

Figure S13. The relationship of different measures of inequality (y-axes) and the number of comments posted before and after each event (x-axes). Colored numbers in each plot correspond to different events (see legend), and patterns are summarized by least-squares fitted lines across events and the Spearman ρ value above each plot.

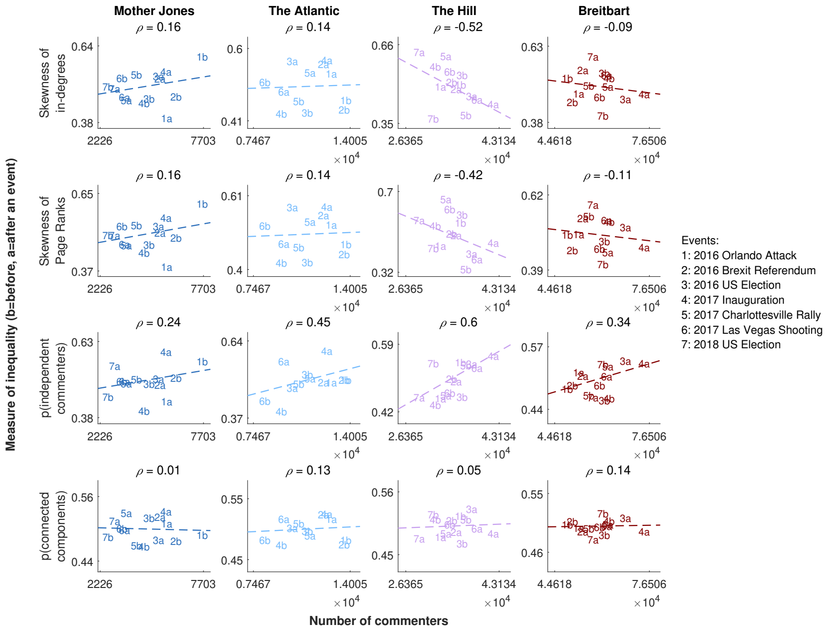

Figure S14. The relationship of different measures of inequality (y-axes) and the number of commenters who participated in the discussions before and after each event (x-axes). Colored numbers in each plot correspond to different events (see legend), and patterns are summarized by least-squares fitted lines across events and the Spearman ρ value above each plot.

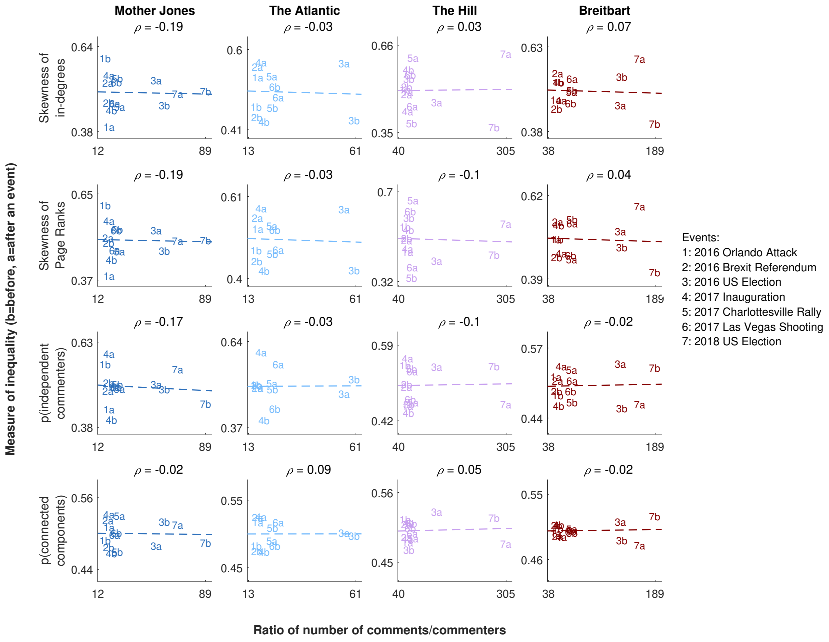

Figure S15. The relationship of different measures of inequality (y-axes) and the ratio of comments to commenters before and after each event (x-axes). Colored numbers in each plot correspond to different events (see legend), and patterns are summarized by least-squares fitted lines across events and the Spearman ρ value above each plot.
